# Supplementary material for: In silico and in vitro evaluation of the anti-virulence potential of patuletin, a natural methoxy flavone, against Pseudomonas aeruginosa
Source: PeerJ. 2024 Feb 1;12:e16826. doi: 10.7717/peerj.16826 (PMC10838535; doi:10.7717/peerj.16826)
Supplement: Supplemental Information 1 [file peerj-12-16826-s001.pdf]

Supplementary data

In silico and in vitro evaluation of the anti-virulance potential of Patuletin, a natural methoxyflavone, against *Pseudomonas aeruginosa*

Ahmed M. Metwaly[a,b], Moustafa M. Saleh[c]*, Aisha A. Alsfouk[d], Ibrahim M. Ibrahim[e], Muhamad Abd-Elraouf[a] , Hazem Elkady[f], Eslam B. Elkaeed[g]*, Ibrahim H. Eissa[f]*

[a] Pharmacognosy and Medicinal Plants Department, Faculty of Pharmacy (Boys), Al-Azhar University, Cairo 11884, Egypt. ametwaly@azhar.edu.eg; mhmraouf@azhar.edu.eg

[b] Biopharmaceutical Products Research Department, Genetic Engineering and Biotechnology Research Institute, City of Scientific Research and Technological Applications (SRTA-City), Alexandria, Egypt.

[c] Microbiology and Immunology Department, Faculty of Pharmacy, Port Said University, Port Said, Egypt. mostafa.mohamed@pharm.psu.edu.eg

[d] Department of Pharmaceutical Sciences, College of Pharmacy, Princess Nourah bint Abdulrahman University, P.O. Box 84428, Riyadh 11671, Saudi Arabia aaalsfouk@pnu.edu.sa;

[e] Biophysics Department, Faculty of Science, Cairo University. Cairo 12613, Egypt. ibrahim_mohamed@cu.edu.eg

[f] Pharmaceutical Medicinal Chemistry & Drug Design Department, Faculty of Pharmacy (Boys), Al-Azhar University, Cairo11884, Egypt. hazemelkady@azhar.edu.eg Ibrahimeissa@azhar.edu.eg

[g] Department of Pharmaceutical Sciences, College of Pharmacy, AlMaarefa University, Riyadh 13713, Saudi Arabia. ekaeed@um.edu.sa

***Corresponding authors:**

Eslam B. Elkaeed **Email:** ekaeed@um.edu.sa
Moustafa M. Saleh **Email:** mostafa.mohamed@pharm.psu.edu.eg

Ibrahim H. Eissa **Email:** Ibrahimeissa@azhar.edu.eg

Molecular Dynamic (MD) Simulation

A standard unbiased MD simulation lasting 100 ns was performed in GROMACS 2021 to assess the stability of the Protein Plateulin complex and to analyze the structural changes that occur between the apo and holo forms of the protein (1). We utilized the solution builder module of the CHARMM-GUI server to generate the input files (2–5). The apo protein and the docked complex were both solvated in an 8.5 nm long cubic box. After solvating the system using the transferable intermolecular potential 3 points (TIP3P) water model with a padding of 1 nm from the furthest distant atom, the system was neutralized by adding NaCl ions at a concentration of 0.154 M. The CHARMM36m force field was used to obtain the amino acid parameters of the protein, as well as those of the TIP3P water model and the neutralizing ions. The Plateulin molecule was parameterized using the CHARMM general force field (CGenFF).

Periodic boundary conditions (PBC) were used in all three dimensions throughout the simulation. To prevent atomic collisions, the potential energy was minimized using the steepest descent algorithm. Following that, the temperature and pressure in the systems were equilibrated in two steps. When the maximum force applied to any atom was less than 100 KJ/(mol.nm), or when the number of reduction steps reached 100,000, the minimizing process was regarded to be converged. The NVT ensemble and the Velocity Rescale technique were utilized to reach an average temperature of 310 K during the first phase of the equilibration process. The NPT ensemble, the Berendsen barostat, and the velocity rescale algorithms were utilized in the second phase to maintain an atmospheric pressure of 1 atm and an average temperature of 310 K (6). For the 100 ns production run, an NPT ensemble was utilized, and the temperature and pressure were regulated by a Nose-Hoover thermostat and a Parrinello-Rahman barostat, respectively. The temperature was kept at 310 degrees Celsius, while the pressure was maintained at one atmospheric pressure (7). The LINear Constraint Solver (LINCS) was used to impose length constraints on the hydrogen-bonded atoms (8).

We utilized Particle Mesh Ewald (PME) to calculate the electrostatics with a threshold of 1.2 nm (9). By using a time step of 1 femtosecond during equilibration and 2 femtoseconds throughout the production run, the Newtonian equations of motion were integrated using the leap-frog approach. We took 1,000 frames at 0.1 ns intervals during the simulation. After the PBC was removed from the system (making the protein whole again) using the trjconv command, we used VMD TK scripts to analyze the trajectory (10). The

RMSDs for Protein and Plateulin were calculated. We also quantified the root mean square fluctuation (RMSF), the radius of gyration (RoG), solvent accessible surface area (SASA), change in hydrogen bond count, and ligand-to-protein center of mass distance to characterize the differences between the apo and holo protein structure and the stability of the compound. Protein-Ligand Interaction Fingerprints (ProLIF) is a Python program that we used to discover which interacting amino acids were most crucial to the binding stability by counting and characterizing the quantity and kind of ligand-amino acid interactions in each frame (11). The protein-ligand complex trajectory was then clustered using TTclust to get representative frames for each cluster. Protein-Ligand interaction profiler (PLIP) was used to determine the number and kind of interactions taking place in these snapshots, and the results were written to a .pse file that can be viewed in PyMol's to observe the 3D conformation of the binding (12,13).

Binding free energy calculation using MM-GBSA:

The binding energy of the ligand was determined using the MM-GBSA strategy using the gmx_MMPBSA software. Decomposition analysis was also used to determine the relative importance of each amino acid within 1 nm of the ligand to the total binding (14,15). A value of 0.154 M for the ionic strength, and a value of 5 for the solvation technique (igb), were chosen. the internal dielectric constant was set to 1.0, whereas it was 78.5 for the external dielectric. The MM-GBSA method can be represented by equation 1.

$$\Delta G = \langle G_{complex} - (G_{receptor} + G_{ligand}) \rangle \qquad \text{Equation 1}$$

Where < > represents the average of the enclosed free energies of complex, receptor, and ligand over the frames used in the calculation. In our approach, we used the part of the trajectory showing stable ligand RMSD (the last 400 frames). Different energy terms can be calculated according to Equations 2 to 6 as follows:

$$\Delta G_{binding} = \Delta H - T\Delta S \qquad \text{Equation 2}$$

$$\Delta H = \Delta E_{gas} + \Delta E_{sol} \qquad \text{Equation 3}$$

$$\Delta E_{gas} = \Delta E_{ele} + \Delta E_{vdW} \qquad \text{Equation 4}$$

$$\Delta E_{solv} = E_{GB} + E_{SA} \qquad \text{Equation 5}$$

$$E_{SA} = \gamma . SASA \qquad \text{Equation 6}$$

Where:

ΔH is the enthalpy which can be calculated from gas-phase energy (E_{gas}) and solvation-free energy (E_{sol}). -TΔS is the entropy contribution to the free binding energy. E_{gas} is composed of electrostatic and van der Waals terms; E_{ele}, E_{vdW}, respectively. E_{sol} can be calculated from the polar solvation energy (E_{GB}) and nonpolar solvation energy (E_{SA}) which is estimated from the solvent-accessible surface area (16,17).

Principal Component Analysis:

Principal component analysis (PCA) of the mass-weighted covariance matrix (C) of atoms in MD trajectories may show coordinated motion. Therefore, we employed principal component analysis to assess the mobility of alpha carbons (18). For the alignment step during studying a single trajectory, the last frame of each trajectory's equilibrium stage was used. The apo system's final frame after it has attained equilibrium was used as the reference structure to examine the combined trajectories. With the aid of the gmx covar command, the C matrix in GROMACS was diagonalized, and the analysis was performed with the assistance of the gmx anaeig command.

The following steps were used to determine the size of the crucial subspace: 1) summing up the eigenvalues with every additional eigenvector (cumulative sum). 2) When plotting eigenvalues at each eigenvector index (scree plot), the point with the steepest decline in slope is selected. 3) Given that non-random eigenvectors do not follow a Gaussian distribution, the distribution of the eigenvectors was studied.

We computed the cosine content (ci) of each eigenvector of the C matrix, which may take values ranging from 0 (no cosine) to 1 (perfect cosine). The following is the cosine content equation:

$$c_i = \frac{2}{T} \left(\int \cos\left(i\pi t\, p_i(t)\, dt\right)^2 \left(\int p_i^2\,(t)\, dt\right)^{-1}$$

Where T is the time of the simulation. Abnormally large ci values, which represent random motion, are related to insufficient sampling. When the cosine content of the first few PCs is near 1, the behavior of proteins on a large scale is analogous to diffusion. Accordingly, the first 10 PCs were used to calculate their cosine content (19–21).

We were able to directly compare the frames in the reduced essential subspace by aligning the combined apo-protein and complex trajectories to the apo-protein configuration obtained after

equilibration, generating a new C matrix for the combined trajectories, and then projecting each trajectory onto the new C matrix.

Bacterial isolates

In the current study, one clinical *P. aeruginosa* isolate (PA1) and a standard *P. aeruginosa* isolate ATCC 27853 (PA27853) were used. The clinical isolate was obtained from the stock culture collection of the Microbiology and Immunology Department at Faculty of Pharmacy, Port Said University, while the standard isolate was a kind gift from Microbiology and Immunology Department, Faculty of Pharmacy, Suez Canal University.

Determination of the minimum inhibitory concentration (MIC) of patuletin

Using the broth microdilution technique in accordance with Clinical and Laboratory Standards Institute (CLSI) procedures (Patel et al., 2015), the MIC of patuletin was assessed. Briefly, a single colony of each of the tested isolates was cultured in Mueller-Hinton broth (MHB) overnight before the bacterial suspensions were diluted using MHB to achieve a cell density of 0.5 McFarland Standard (10^7 CFU/ml). The tested substance, patuletin, was utilized to prepare two-fold serial dilution solutions. On microtiter plates, 100 µl of the tested bacterial suspensions were mixed with 100 µl of the prepared diluted tested substance. The lowest concentration of the tested substance that prevented bacterial growth that was observable after an overnight incubation at 37°C was determined to be the MIC value.

Evaluation of patuletin impact on virulence factors production in *P. aeruginosa*

Biofilm inhibition assay

According to Stepanovi et al. (2007) procedures, the ability of the tested isolates to generate biofilms was evaluated. *P. aeruginosa* suspensions were prepared from cultures that had been grown in tryptone soy broth (TSB) overnight and then diluted to the 0.5 McFarland standard. The prepared suspensions were diluted 1:100 in new TSB with 1% glucose addition. The wells of microtiter plates were filled with aliquots of 200 µl of diluted suspensions, which were then incubated for 48 hours at 37°C in the presence and absence of sub-MIC of patuletin. Each plate

included negative-control wells that contained 200 µl of fresh TSB supplemented with 1% glucose alone. After incubation, the well contents were decanted, and the plates underwent three water washes to remove any remaining planktonic cells before being allowed to air dry. To fix the biofilms, 150 µl of 99% methanol were applied in aliquots and left for 20 min. The biofilms were then stained for 15 minutes with aliquots of 150 µl of crystal violet (1%) before being rinsed three times with water and dried. The bound dye was solubilized with 150 µl of 33% glacial acetic acid. The OD_{570} was measured using spectrofluorometer (Biotek, USA). The experiment was done in triplicate. The biofilm forming capacity was assessed according to the criteria of Stepanović et al. (2007). From the following formula, the percentage (%) of biofilm inhibition was calculated:

Percentage of biofilm inhibition = $[(Control_{OD570nm} - Treated_{OD570nm})/ Control_{OD570nm} \times 100$

Pyocyanin inhibition assay

Pyocyanin estimation was carried out following Das and Manefield (2012) procedures. Using overnight cultures of the tested isolates in Luria-Bertani (LB) broth that had been diluted to an OD_{600} of 0.3–0.4, pyocyanin was quantified. In both the presence and absence of sub-MIC of patuletin, 50 µl of the diluted cultures of each isolate were added to 5 ml of LB broth. The cultures were centrifuged at 10,000 rpm for 10 minutes at 4°C after being incubated at 37°C for 48 hours and the pyocyanin pigment in the supernatants was measured directly at 691 nm using spectrofluorometer (Biotek, USA).

Proteases inhibition assay

According to El-Mowafy et al. 2014, the modified skim milk technique was used to assess the patuletin ability to suppress the production of proteases. In brief, *P. aeruginosa* isolates were cultured overnight in LB broth with and without sub-MIC of patuletin. Supernatants were produced by centrifuging bacterial suspensions at 10,000 rpm for 10 minutes. The next step was to mix 0.5 ml of each tested isolate cell-free supernatant with 1 ml of skim milk solution (1.25% in distilled water), which was then incubated for ½ hr at 37 °C. Using a spectrofluorometer (Biotek, USA) the turbidities of assay solutions were assessed at OD_{600} as a measure of proteolytic activity.

Statistical analysis

The GraphPad Prism 7 software package was used to analyze the data for the current study. The effect of patuletin on *P. aeruginosa* virulence factors was compared using One Way ANOVA at $P > 0.05$ for significance. The results were computed using the means and standard errors of three biological tests with three technical replicates.

Biofilm inhibition assay readings and results

Inhibitors / Isolate No	Control (untreated) isolates absorbance (OD_{570})	Average Control (untreated) isolates absorbance (OD_{570}))	Patuletin treated isolates absorbance (biofilm formation ability)	Average Patuletin treated isolates absorbance (% biofilm formation ability)	Average % reduction
**PA 27853**	R1= 0.405	0.385	R1= 0.180	0.185 (48%)	52%
	R2= 0.370		R2= 0.200		
	R3= 0.385		R3= 0.175		
**PA 1**	R1= 0.474	0.456	R1= 0.187	0.192 (42%)	58%
	R2= 0.460		R2= 0.196		
	R3= 0.434		R3= 0.193		

References

1. Abraham MJ, Murtola T, Schulz R, Páll S, Smith JC, Hess B, et al. GROMACS: High performance molecular simulations through multi-level parallelism from laptops to supercomputers. SoftwareX. 2015;1:19–25.

2. Brooks BR, Brooks III CL, Mackerell Jr AD, Nilsson L, Petrella RJ, Roux B, et al. CHARMM: the biomolecular simulation program. J Comput Chem. 2009;30(10):1545–614.

3. Jo S, Cheng X, Islam SM, Huang L, Rui H, Zhu A, et al. Chapter Eight - CHARMM-GUI PDB Manipulator for Advanced Modeling and Simulations of Proteins Containing Nonstandard Residues. In: Karabencheva-Christova T, editor. Biomolecular Modelling and Simulations. Academic Press; 2014. p. 235–65. (Advances in Protein Chemistry and Structural Biology; vol. 96).

4. Jo S, Kim T, Iyer VG, Im W. CHARMM-GUI: A web-based graphical user interface for CHARMM. J Comput Chem. 2008;29(11):1859–65.

5. Lee J, Cheng X, Swails JM, Yeom MS, Eastman PK, Lemkul JA, et al. CHARMM-GUI input generator for NAMD, GROMACS, AMBER, OpenMM, and CHARMM/OpenMM simulations using the CHARMM36 additive force field. J Chem Theory Comput. 2016;12(1):405–13.

6. Bussi G, Donadio D, Parrinello M. Canonical sampling through velocity rescaling. J Chem Phys. 2007;126(1):14101.

7. Evans DJ, Holian BL. The nose–hoover thermostat. J Chem Phys. 1985;83(8):4069–74.

8. Hess B, Bekker H, Berendsen HJC, Fraaije JGEM. LINCS: a linear constraint solver for molecular simulations. J Comput Chem. 1997;18(12):1463–72.

9. Essmann U, Perera L, Berkowitz ML, Darden T, Lee H, Pedersen LG. A smooth particle mesh Ewald method. J Chem Phys. 1995;103(19):8577–93.

10. Humphrey W, Dalke A, Schulten K. VMD: visual molecular dynamics. J Mol Graph. 1996;14(1):33–8.

11. Bouysset C, Fiorucci S. ProLIF: a library to encode molecular interactions as fingerprints. J Cheminform. 2021;13(1):1–9.

12. Salentin S, Schreiber S, Haupt VJ, Adasme MF, Schroeder M. PLIP: fully automated protein–ligand interaction profiler. Nucleic Acids Res. 2015;43(W1):W443–W447.

13. Tubiana T, Carvaillo JC, Boulard Y, Bressanelli S. TTClust: a versatile molecular simulation trajectory clustering program with graphical summaries. J Chem Inf Model. 2018;58(11):2178–82.

14. Valdés-Tresanco MS, Valdés-Tresanco ME, Valiente PA, Moreno E. gmx_MMPBSA: A New Tool to Perform End-State Free Energy Calculations with GROMACS. J Chem Theory Comput. 2021;17(10):6281–91.

15. Miller III BR, McGee Jr TD, Swails JM, Homeyer N, Gohlke H, Roitberg AE. MMPBSA. py: an efficient program for end-state free energy calculations. J Chem Theory Comput. 2012;8(9):3314–21.

16. Xue W, Yang F, Wang P, Zheng G, Chen Y, Yao X, et al. What contributes to serotonin--norepinephrine reuptake inhibitors' dual-targeting mechanism? The key role of transmembrane domain 6 in human serotonin and norepinephrine transporters revealed by molecular dynamics simulation. ACS Chem Neurosci. 2018;9(5):1128–40.

17. Tuccinardi T. What is the current value of MM/PBSA and MM/GBSA methods in drug discovery? Vol. 16, Expert opinion on drug discovery. Taylor \& Francis; 2021. p. 1233–7.

18. Amadei A, Linssen ABM, Berendsen HJC. Essential dynamics of proteins. Proteins: Structure, Function, and Bioinformatics. 1993;17(4):412–25.

19. Papaleo E, Mereghetti P, Fantucci P, Grandori R, de Gioia L. Free-energy landscape, principal component analysis, and structural clustering to identify representative conformations from molecular dynamics simulations: the myoglobin case. J Mol Graph Model. 2009;27(8):889–99.

20. Maisuradze GG, Leitner DM. Free energy landscape of a biomolecule in dihedral principal component space: Sampling convergence and correspondence between structures and minima. Proteins: Structure, Function, and Bioinformatics. 2007;67(3):569–78.

21. Hess B. Similarities between principal components of protein dynamics and random diffusion. Phys Rev E. 2000;62(6):8438.
